# Clinical significance of angiopoietin-like protein 4 expression in tissue and serum of esophageal squamous cell carcinoma patients

**DOI:** 10.1007/s12032-013-0680-y

**Published:** 2013-08-08

**Authors:** Jun Yi, Ban-zhou Pan, Lei Xiong, Hai-zhu Song

**Affiliations:** 1 Department of Cardiothoracic Surgery, Jinling Hospital, Medical School of Nanjing University, Nanjing, 210002 China; 2 Department of Medical Oncology, Jinling Hospital, Medical School of Nanjing University, Nanjing, 210002 China

**Keywords:** Esophageal squamous cell carcinoma, Angiopoietin-like protein 4, Diagnosis, Prognosis

## Abstract

Angiopoietin-like protein 4 (ANGPTL4) has been reported to promote tumor growth, metastasis, and angiogenesis under certain conditions. The aim of this study was to examine ANGPTL4 expression in tumor and serum tissues from esophageal squamous cell carcinoma (ESCC) patients. A total of 78 ESCC patients treated with radical resection were enrolled in this study. Immunohistochemistry was used to detect ANGPTL4 expression in ESCC tissues. Serum ANGPTL4 levels were determined via enzyme-linked immunosorbent assay (ELISA). The receiver operating characteristics curve was constructed to describe diagnostic specificity and sensitivity. There were 52 cases (69.2 %) showing a higher level of ANGPTL4 expression in tumor tissues than that in normal tissues, and the rate of ANGPTL4 protein high/moderate expression in ESCC and normal tissues was 55.1 % (43/78) and 6.4 % (5/78), respectively, with a significant difference (*P* < 0.001). Moreover, the high/moderate of ANGPTL4 protein was significantly associated with lymph metastasis, clinical stage, and adverse 2-year progression-free survival. In addition, serum ANGPTL4 level in ESCC patients was much higher than that in patients with benign esophageal disease (*P* < 0.001), and area under the curve was 0.94 (95 % CI 0.886390–0.978173, *P* < 0.001). But serum ANGPTL4 level was significantly decreased at post-operative 7–10 days (*P* = 0.004). ANGPTL4 upregulation may play an important role in ESCC development, and serum ANGPTL4 level may be a potential tumor marker for ESCC diagnosis and prognosis.

## Background

Esophageal squamous cell carcinoma (ESCC) comprises the majority of esophageal cancer in China and is characterized by both high morbidity and high mortality [1]. Although the disease is surgically curable in its early stages, patients often suffer asymptomatic metastasis that is associated with a high mortality [2]. Therefore, it is crucial to develop more effective screening methods to better predict the course of the disease. Currently, the most important conventional prognostic factors are the histological grade and tumor stage at the time of diagnosis (pTNM). In addition, molecular markers such as circulating tumor cells, microRNAs, and DNA methylation are also employed [3], contributing to improving the diagnosis, prognosis, and guidance of adjuvant treatments for ESCC.

Angiopoietin-like protein 4 (ANGPTL4) is a member of the angiopoietin (ANG) family, which encodes a secretory glycoprotein highly expressed in adipose tissue, liver, placental tissue, and ischemic tissues [4]. ANGPTL4 was previously identified as a paracrine and, possibly, endocrine regulator of lipid metabolism and a target of peroxisome proliferators-activated receptors (PPARs) [5]. Recently, emerging evidence has identified a novel role for ANGPTL4 in cancer development [6]. Several studies suggest that ANGPTL4 can prevent metastasis by inhibiting vascular leakiness [7, 8]. Conversely, ANGPTL4 is also implicated as a pro-angiogenic factor [4], and tumor-derived ANGPTL4 has been shown to promote metastasis by disrupting vascular integrity [9]. The explanation of these conflicting results and the underlying mechanism of ANGPTL4 activity in tumor cells have not been fully clarified. Recent reports demonstrate that ANGPTL4 expression is upregulated in the majority of human cancers including ESCC [6, 10]. As a secreted protein, elevated levels ANGPTL4 protein have also been detected in the serum of hepatocellular carcinoma (HCC) and tumor-bearing mice [11, 12]. Therefore, to explore the precise role of ANGPTL4 in ESCC pathogenesis, we detected ANGPTL4 expression levels in tissue and serum samples from ESCC patients treated with radical resection.

## Methods

### Study population

This study enrolled 78 ESCC patients who underwent radical resection without neoadjuvant treatment at Jinling Hospital from July 2006 to July 2008. All patients underwent en bloc esophagectomy with locoregional lymphadenectomy through a right thoracotomy, laparotomy with reconstruction using the stomach through a retrosternal route, and cervical esophagogastrostomy. For patients at stage IIb or beyond, concurrent or sequential chemoradiotherapy was administered after surgery.

Patients consisted of 50 males and 28 females, with a median age of 62 (range 44–83) years. Tumor stage was conducted according to the 7th edition of the TNM staging system of the International Union Against Cancer [13], and patients were at stages I (*n* = 15), II (*n* = 25), III (*n* = 33), and IV (*n* = 5, supraclavicular or para-aortic lymph nodes metastasis). Cellular differentiation was graded according to the WHO grading system. Ethical approval was obtained from the hospital, and informed consent was obtained from all patients prior to sample examination. Clinical follow-up data were available for all the patients. For each patient, 5 mL peripheral blood pre-operation and post-operation (7–10 days) was collected by promoting coagulation tubes, and then, serum was isolated. Serum samples from 40 patients with benign esophageal disease (10 cases of chronic esophagitis, 10 cases of esophageal leiomyoma, 10 cases of esophageal polyps, and 10 cases of esophageal achalasia) were also collected.

### Immunohistochemical staining

Formalin-fixed, paraffin-embedded samples used for immunohistochemistry were sectioned at 2 μm thickness. Sections were deparaffinized using xylene, dehydrated by gradient ethanol, and then rehydrated with deionized water. Heat-mediated antigen retrieval was run by autoclave treatment (120 °C for 2 min in 1 mmol/L ethylenediaminetetraacetic acid [EDTA], pH 8.0) and then followed by cooling at room temperature. Incubation with a polyclonal rabbit anti-ANGPTL4 antibody (diluted 1:150; R&D Systems, Minneapolis, Minnesota, USA) was performed overnight at 4 °C. After washing with phosphate-buffered saline (PBS), sections were then incubated with goat anti-rabbit secondary antibody (Santa Cruz Biotechnology, CA, USA) for 30 min at room temperature. Coloration was performed with 3,3-diaminobenzidine. Nuclei were counterstained with hematoxylin. PBS was used as a negative control for the staining reactions. Immunostaining results were evaluated independently by 3 pathologists. The percentage of positive cells was rated as follows: 0 score for 0–5 %, 1 score for 6–25 %, 2 scores for 26–50 %, and 3 scores for more than 50 %. The staining intensity was rated as follows: 0 score for no staining, 1 score for weak staining, 2 scores for moderate staining, and 3 scores for strong staining [14]. The scores from the percentage and intensity were added to an overall score, and the expression of the ANGPTL4 protein with an overall score of 0–2 was designated as ‘low/negative,’ and with an overall score of 3–6 was designated as ‘high/moderate.’

### ANGPTL4 determination in serum

Serum ANGPTL4 levels were determined via enzyme-linked immunosorbent assay (ELISA) in duplicate, using the DuoSet ELISA hANGPTL4 kit (R&D Systems, Minneapolis, MN) according to the manufacturer’s instructions. The serum samples were diluted 1:10 in PBS prior to detection.

### Statistical analysis

Statistical tests were carried out using SPSS version 16.0 (SPSS Inc., Chicago, IL, USA). The differences of ANGPTL4 expression between the groups were calculated with Student’s *t* test. Differences in frequency were assessed by chi-square test. Overall survival curves were calculated using the Kaplan–Meier method and compared by log-rank testing. Multivariate Cox proportional hazard models were used to define the potential prognostic significance of individual parameter. The receiver operating characteristics (ROC) curve was constructed to describe diagnostic specificity and sensitivity. *P* < 0.05 was taken as statistically significant.

## Results

### ANGPTL4 protein expression profiles in ESCC tissue

**Fig. 1:**
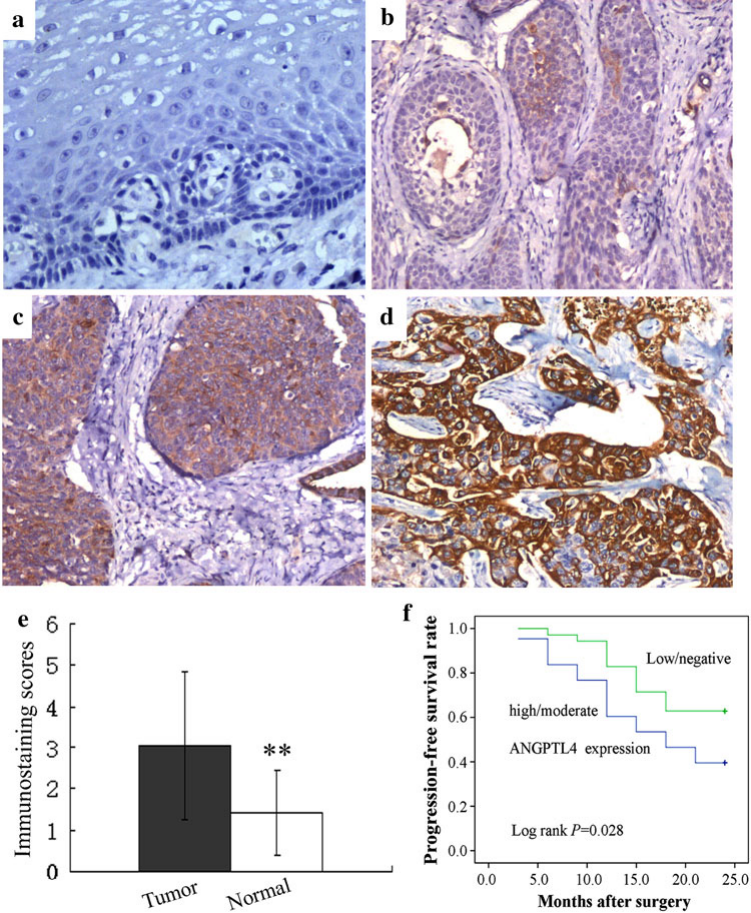
Increased ANGPTL4 expression in ESCC tissues determined by immunohistochemical staining (EnVision, magnification ×400). **a** Negative in adjacent normal esophagus tissues; **b** low expression of ANGPTL4 in tumor; **c** moderate expression of ANGPTL4 in tumor; **d** high expression of ANGPTL4 in tumor; **e** the average immunostaining scores of ANGPTL4 expression in tumor and normal tissues; **f** 2-year progression-free survival (PFS) was analyzed by Kaplan–Meier survival curve. *P < 0.05

We detected ANGPTL4 protein expression levels in 78 pairs of ESCC and matched normal tissues by immunohistochemical staining. The representative immunohistochemical results are shown in Fig. 1a–d. In total, there were (delete) 52 cases (69.2 %) showed a higher level of ANGPTL4 protein expression in tumor tissues than that in normal tissues, with the average immunostaining scores in tumor tissues being 3.04 ± 1.80 and 1.42 ± 1.01 in normal tissues (Fig. 1e, *P* < 0.001). Moreover, the rate of ANGPTL4 protein high/moderate expression in ESCC and normal tissues was 55.1 % (43/78) and 6.4 % (5/78), respectively, which showed a significant difference (*P* < 0.001).

### Association between ANGPTL4 protein expression and clinicopathological features

As shown in Table 1, an elevated ANGPTL4 protein expression in ESCC tissue was significantly associated with lymph metastasis and clinical stage. However, there was no correlation between ANGPTL4 protein expression and patients’ gender, age, tumor site, depth or cellular differentiation.

**Table 1:** Association between ANGPTL4 expression in ESCC tissues and clinicopathological features

Characteristics	No.	High/moderate expression of ANGPTL4 n (%)	*P* value
Gender			
Male	50	29 (58.0)	0.469
Female	28	14 (50.0)	
Age			
<60	32	15 (46.9)	0.222
≥60	46	28 (60.9)	
Tumor site			
Upper thoracic	15	7 (46.7)	0.646
Middle thoracic	30	16 (53.3)	
Lower thoracic	33	20 (60.6)	
T status			
T1–2	42	20 (47.6)	0.150
T3–4	36	23 (63.9)	
Differentiation			
Well	14	6 (42.9)	0.322
Moderate	36	23 (63.9)	
Poor	28	14 (50.0)	
Stage			
I/II	40	17 (42.5)	0.021*
III/IV	38	26 (68.4)	
Lymph			
N_0_	36	15 (41.7)	0.027*
N_1_/N_2_/N_3_	42	28 (66.7)	

* *P* < 0.05

### Association between ANGPTL4 protein expression and ESCC prognosis

To the follow-up deadline, there were 39 patients with progression or relapse within 2 years after the end of surgery. We performed univariate survival analyses to investigate the possible prognostic role of ANGPTL4 expression in ESCC. As shown in Fig. 1f, the 2-year progression-free survival (PFS) in ESCC patients with high/moderate expression of ANGPTL4 protein was inferior to that with low/negative expression [mean 16.7 months (95 % CI 14.510–18.839) vs. 20.1 months (95 % CI 18.340–21.946), *P* = 0.028].

Furthermore, multiple Cox regression analysis was used to verify whether the investigated variables including ANGPTL4 expression were valid predictors of outcome after adjusting for potential confounding cofactors. Results showed that high/moderate expression of ANGPTL4 protein, apart from lymph metastasis, was independent factor for predicting an adverse 2-year PFS for ESCC patients (Table 2).

**Table 2:** Multivariate analysis of clinicopathological factors for 2-year progression-free survival (PFS) of 78 patients with ESCC

Characteristics	Category	RR (95 % CI)	*P* value
Age	≥60 versus <60 years	1.523 (0.603–3.812)	0.389
Tumor differentiation	Poor versus well/moderate	1.656 (0.611–4.109)	0.305
T status	T3–4 versus T1–2	1.751 (0.784–3.921)	0.172
Lesion length	≥5 cm versus <5 cm	1.172 (0.435–3.156)	0.652
Lymph metastasis	N_1_/N_2_/N_3_ versus N_0_	2.845 (1.132–7.212)	0.025*
ANGPTL4 expression in tissue	High/moderate versus low/negative	2.594 (1.016–6.568)	0.034*
KPS scores	≥90 versus <90	0.585 (0.223–1.620)	0.423

* *P* < 0.05

### Serum ANGPTL4 levels in pre-/post-operative ESCC patients

**Fig. 2:**
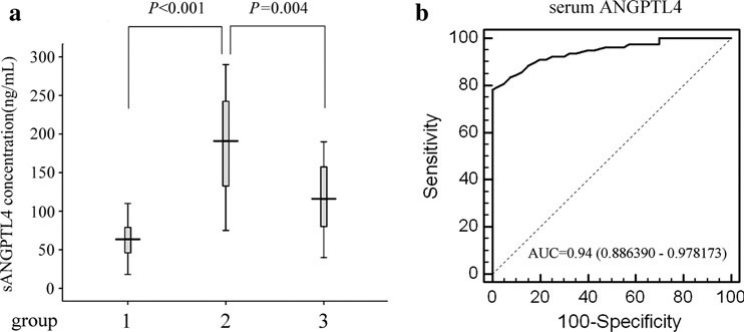
Serum ANGPTL4 levels in ESCC patients and controls. **a** Serum ANGPTL4 levels determined by ELISA. The data are expressed as mean ± SD, group 1, patients with benign esophageal disease as controls (*n* = 40); group 2, pre-operative ESCC patients (*n* = 78); group 3, post-operative ESCC patients (n = 78). **b** ROC curve was constructed to describe the diagnostic specificity and sensitivity of serum ANGPTL4 in pre-operative ESCC patients and controls

As shown in Fig. 2a, serum ANGPTL4 level in ESCC patients was significantly higher than that in patients with benign esophageal disease (202.44 ± 131.03 vs. 63.19 ± 23.06 ng/mL, *P* < 0.001). Furthermore, serum ANGPTL4 levels in most ESCC patients were decreased after 7–10 days of surgery, and the pre-/post-operative concentrations were 202.44 ± 131.03 ng/mL and 128.73 ± 69.49 ng/mL, respectively (*P* = 0.004).

We then constructed ROC curve to describe the diagnostic specificity and sensitivity of serum ANGPTL4. Obviously, its detectability to distinguish malignant tumor is satisfactory, area under the curve (AUC) was 0.94 (95 % CI 0.886390–0.978173, *P* < 0.001), and Youden index was 0.78 (Fig. 2b).

## Discussion

Cancer cells do not exist in isolation but rather within a complex milieu of normal cells, secreted proteins, physiological small molecules, and blood vessels which comprise the tumor microenvironment [15]. ANGPTL4 is a novel secretory glycoprotein which has been reported to interact with specific extracellular matrix (ECM) proteins and integrins to facilitate cell migration during wound healing and cancer development [16, 17]. But the literature regarding the functions of ANGPTL4 is contradictory. Both in vitro and in vivo data suggest that ANGPTL4 has pro-angiogenic effects [4]. ANGPTL4 could stimulate endothelial cell growth and tubule formation [18], while the inhibition of ANGPTL4 using siRNAs prevented the neovascularization process and reduced vascular permeability [19]. Moreover, ANGPTL4 has been reported to promote tumor growth [20], help tumor cells avoid anoikis [12, 21], and facilitate tumor cell metastases by interfering with endothelial cell junctions and induction of vascular leakiness [9, 22, 23]. Furthermore, clinical studies have correlated ANGPTL4 expression with venous and lymphatic invasion in human gastric and colorectal cancers, which further emphasized the role of ANGPTL4 in tumor metastasis [24, 25]. The elevated expression of ANGPTL4 was also proved to be correlated with poor prognosis in esophageal and tongue cancers [10, 26]. On the other hand, several studies showed that increased ANGPTL4 expression could inhibit tumor growth, metastasis, and angiogenesis in melanoma, lung, and colorectal cancers [7, 8]. Recently, Okochi-Takada et al. [27] found that ANGPTL4 was a genetically and epigenetically inactivated secreted tumor suppressor and inhibited tumor angiogenesis in gastric cancer. These results suggest that the expression and roles of ANGPTL4 may be tumor-type and microenvironment dependent.

In concordance with the results of Shibata et al. [10], we found that the level of ANGPTL4 protein expression in ESCC was much higher than that in matched normal tissues, and an elevated expression of ANGPTL4 protein was significantly associated with lymph metastasis, clinical stage, and adverse 2-year PFS, further confirming the important roles of ANGPTL4 dysfunction in ESCC development in Chinese population.

The expression of ANGPTL4 is considered to be regulated by the nuclear hormone receptors of the PPAR family, as well as by hypoxia and chronic inflammatory pressure. A recent study suggested that prostaglandin E2 (PGE2) may transactivate PPARβ/δ to regulate the expression of ANGPTL4 under hypoxic conditions [20]. Because of the aberrant growth of tumor cells and poor vascularization, the tumor microenvironment tends to become hypoxic. Several new studies have reported that hypoxia can induce the expression of ANGPTL4 and that upregulation of ANGPTL4 is induced by the transcription factor hypoxia-inducible factor 1α (HIF-1α) [11, 20, 28]. Moreover, the potent regulator of inflammation TGF-β was found to regulate the expression of ANGPTL4 via a Smad3 signaling pathway [9]. Therefore, we supposed that upregulation of ANGPTL4 in ESCC was an adaptive response to tumor microenvironment, which in turn influence tumor development. For example, this increase in ANGPTL4 expression enhanced endothelial cell migration and differentiation, both of which are important processes in angiogenesis [19], suggesting that it may play a role in the metastatic processes. Furthermore, studies have pinpointed ANGPTL4 as a critical mediator in the transmigration process [12, 22]. TGF-β upregulated ANGPTL4 in breast tumor cells and then disrupted the endothelial cell–cell junctions, leading to enhanced vascular leakiness and transendothelial migration of the tumor cells [9]. ANGPTL4 was found to promote transendothelial migration and metastasis of HCC cells through the upregulation of vascular cell adhesion molecule 1 (VCAM-1) on endothelial cells [11].

In clinical practice, the actual outcome of the patients is not entirely consistent with the clinicopathological parameters such as lesion length, invasion depth, and lymph metastasis, a frequently updated pTNM still fails to discriminate between degrees of malignancy. One at an early stage suffers tumor recurrence or metastasis soon after initial treatment, and others at advanced stages have long-term survival [29], probably due to the different molecular biology characteristics of the tumors. Molecular markers are being sought in ESCC, but few can be applied in the peripheral blood detection. In the present study, we found that serum ANGPTL4 level in ESCC patients was significantly lower than that in patients with benign esophageal disease, and its diagnostic efficacy was satisfactory according to AUC curve. Furthermore, serum ANGPTL4 levels in most ESCC patients were decreased after 7–10 days after surgery. These results suggested that serum ANGPTL4 level may be a sensitive tumor biomarker for diagnosing or monitoring ESCC.

Previous studies have also shown that serum ANGPTL4 was significantly increased in HCC patients, compared with chronic hepatitis B patients and normal controls, and ANGPTL4 levels were significantly higher in HCC patients with intrahepatic metastasis and macrovascular invasion than those without [11]. However, the data in colorectal cancer were diametrically opposed, and Kumara et al. [30] believe that ANGPTL4 decrease after surgery must be related to the surgical trauma itself or to the body’s response to that insult. Thus, serum ANGPTL4 may not be exclusively secreted by tumor cells, and its application in clinic needs further study. In our study, whether the serum ANGPTL4 decrease after ESCC surgery is due to tumor load reduction, or its raised level in a certain period be related to tumor recurrence or progression should be validated by long-term follow-up data in the future.

## Conclusions

We confirmed that ANGPTL4 upregulation plays important roles in ESCC development, and serum ANGPTL4 level may be a potential tumor marker for ESCC diagnosis and prognosis.

## Abbreviations

ANGPTL4: Angiopoietin-like protein 4
AUC: Area under the curve
ELISA: Enzyme-linked immunosorbent assay
ESCC: Esophageal squamous cell carcinoma
HCC: Hepatocellular carcinoma
HIF-1α: Hypoxia-inducible factor 1α
PPARs: Proliferators-activated receptors

## References

[1] Zheng S, Vuitton L, Sheyhidin I, Vuitton DA, Zhang Y, Lu X (2010). Northwestern China: a place to learn more on oesophageal cancer. Part one: behavioural and environmental risk factors. Eur J Gastroenterol Hepatol.

[2] Bagheri R, Maddah G, Saedi HS, Sadeghian MH, Roodbari S (2011). Bone marrow involvement in esophageal cancer patients who underwent surgical resection. Eur J Cardiothorac Surg.

[3] Zheng Y, Zhang Y, Huang X, Chen L (2011). Analysis of the RUNX3 gene methylation in serum DNA from esophagus squamous cell carcinoma, gastric and colorectal adenocarcinoma patients. Hepatogastroenterology.

[4] Le Jan S, Amy C, Cazes A, Monnot C, Lamandé N, Favier J, Philippe J, Sibony M, Gasc JM, Corvol P, Germain S (2003). Angiopoietin-like 4 is a proangiogenic factor produced during ischemia and in conventional renal cell carcinoma. Am J Pathol.

[5] Sukonina V, Lookene A, Olivecrona T, Olivecrona G (2006). Angiopoietin-like protein 4 converts lipoprotein lipase to inactive monomers and modulates lipase activity in adipose tissue. Proc Natl Acad Sci USA.

[6] Tan MJ, Teo Z, Sng MK, Zhu P, Tan NS (2012). Emerging roles of angiopoietin-like 4 in human cancer. Mol Cancer Res.

[7] Galaup A, Cazes A, Le Jan S, Philippe J, Connault E, Le Coz E, Mekid H, Mir LM, Opolon P, Corvol P, Monnot C, Germain S (2006). Angiopoietin-like 4 prevents metastasis through inhibition of vascular permeability and tumor cell motility and invasiveness. Proc Natl Acad Sci USA.

[8] Ito Y, Oike Y, Yasunaga K, Hamada K, Miyata K, Matsumoto S, Sugano S, Tanihara H, Masuho Y, Suda T (2003). Inhibition of angiogenesis and vascular leakiness by angiopoietin-related protein 4. Cancer Res.

[9] Padua D, Zhang XH, Wang Q, Nadal C, Gerald WL, Gomis RR, Massagué J (2008). TGFbeta primes breast tumors for lung metastasis seeding through angiopoietin-like 4. Cell.

[10] Shibata K, Nakayama T, Hirakawa H, Hidaka S, Nagayasu T (2010). Clinicopathological significance of angiopoietin-like protein 4 expression in oesophageal squamous cell carcinoma. J Clin Pathol.

[11] Li H, Ge C, Zhao F, Yan M, Hu C, Jia D, Tian H, Zhu M, Chen T, Jiang G, Xie H, Cui Y, Gu J, Tu H, He X, Yao M, Liu Y, Li J (2011). Hypoxia-inducible factor 1 alpha-activated angiopoietin-like protein 4 contributes to tumor metastasis via vascular cell adhesion molecule-1/integrin β1 signaling in human hepatocellular carcinoma. Hepatology.

[12] Zhu P, Tan MJ, Huang RL, Tan CK, Chong HC, Pal M, Lam CR, Boukamp P, Pan JY, Tan SH, Kersten S, Li HY, Ding JL, Tan NS (2011). Angiopoietin-like 4 protein elevates the prosurvival intracellular O_2_(-):H_2_O_2_ ratio and confers anoikis resistance to tumors. Cancer Cell.

[13] Rice TW, Blackstone EH, Rusch VW (2010). 7th edition of the AJCC cancer staging manual: esophagus and esophagogastric junction. Ann Surg Oncol.

[14] Song H, Xu B, Yi J (2012). Clinical significance of stanniocalcin-1 detected in peripheral blood and bone marrow of esophageal squamous cell carcinoma patients. J Exp Clin Cancer Res.

[15] Gao D, Vahdat LT, Wong S, Chang JC, Mittal V (2012). Microenvironmental regulation of epithelial-mesenchymal transitions in cancer. Cancer Res.

[16] Cazes A, Galaup A, Chomel C, Bignon M, Bréchot N, Le Jan S, Weber H, Corvol P, Muller L, Germain S, Monnot C (2006). Extracellular matrix-bound angiopoietin-like 4 inhibits endothelial cell adhesion, migration, and sprouting and alters actin cytoskeleton. Circ Res.

[17] Goh YY, Pal M, Chong HC, Zhu P, Tan MJ, Punugu L, Lam CR, Yau YH, Tan CK, Huang RL, Tan SM, Tang MB, Ding JL, Kersten S, Tan NS (2010). Angiopoietin-like 4 interacts with integrins beta1 and beta5 to modulate keratinocyte migration. Am J Pathol.

[18] Gealekman O, Burkart A, Chouinard M, Nicoloro SM, Straubhaar J, Corvera S (2008). Enhanced angiogenesis in obesity and in response to PPARgamma activators through adipocyte VEGF and ANGPTL4 production. Am J Physiol Endocrinol Metab.

[19] Ma T, Jham BC, Hu J, Friedman ER, Basile JR, Molinolo A, Sodhi A, Montaner S (2010). Viral G protein-coupled receptor up-regulates angiopoietin-like 4 promoting angiogenesis and vascular permeability in Kaposi’s sarcoma. Proc Natl Acad Sci USA.

[20] Kim SH, Park YY, Kim SW, Lee JS, Wang D, DuBois RN (2011). ANGPTL4 induction by prostaglandin E2 under hypoxic conditions promotes colorectal cancer progression. Cancer Res.

[21] Terada LS, Nwariaku FE (2011). Escaping Anoikis through ROS: ANGPTL4 controls integrin signaling through Nox1. Cancer Cell.

[22] Huang RL, Teo Z, Chong HC, Zhu P, Tan MJ, Tan CK, Lam CR, Sng MK, Leong DT, Tan SM, Kersten S, Ding JL, Li HY, Tan NS (2011). ANGPTL4 modulates vascular junction integrity by integrin signaling and disruption of intercellular VE-cadherin and claudin-5 clusters. Blood.

[23] Huang XF, Han J, Hu XT, He C (2012). Mechanisms involved in biological behavior changes associated with Angptl4 expression in colon cancer cell lines. Oncol Rep.

[24] Nakayama T, Hirakawa H, Shibata K, Abe K, Nagayasu T, Taguchi T (2010). Expression of angiopoietin-like 4 in human gastric cancer: ANGPTL4 promotes venous invasion. Oncol Rep.

[25] Nakayama T, Hirakawa H, Shibata K, Nazneen A, Abe K, Nagayasu T, Taguchi T (2011). Expression of angiopoietin-like 4 (ANGPTL4) in human colorectal cancer: ANGPTL4 promotes venous invasion and distant metastasis. Oncol Rep.

[26] Wang Z, Han B, Zhang Z, Pan J, Xia H (2010). Expression of angiopoietin-like 4 and tenascin C but not cathepsin C mRNA predicts prognosis of oral tongue squamous cell carcinoma. Biomarkers.

[27] Okochi-Takada E, Hattori N, Tsukamoto T, Miyamoto K, Ando T, Ito S, Yamamura Y, Wakabayashi M, Nobeyama Y, Ushijima T. ANGPTL4 is a secreted tumor suppressor that inhibits angiogenesis. Oncogene 2013. doi:10.1038/onc.2013.174. [Epub ahead of print]. 10.1038/onc.2013.174 23686315

[28] Zhang H, Wong CC, Wei H, Gilkes DM, Korangath P, Chaturvedi P, Schito L, Chen J, Krishnamachary B, Winnard PT, Raman V, Zhen L, Mitzner WA, Sukumar S, Semenza GL (2012). HIF-1-dependent expression of angiopoietin-like 4 and L1CAM mediates vascular metastasis of hypoxic breast cancer cells to the lungs. Oncogene.

[29] Okamura S, Fujiwara H, Shiozaki A, Komatsu S, Ichikawa D, Okamoto K, Murayama Y, Ikoma H, Kuriu Y, Nakanishi M, Ochiai T, Kokuba Y, Sonoyama T, Otsuji E (2011). Long-term survivors of esophageal carcinoma with distant lymph node metastasis. Hepatogastroenterology.

[30] Kumara HM, Kirchoff D, Herath SA, Jang JH, Yan X, Grieco M, Cekic V, Whelan RL (2012). Plasma levels of angiopoietin-like protein 4 (ANGPTL4) are significantly lower preoperatively in colorectal cancer patients than in cancer-free patients and are further decreased during the first month after minimally invasive colorectal resection. Surg Endosc.

